# AgeSpectraAnalyst: A MATLAB based package to model zircon age distributions in silicic magmatic systems

**DOI:** 10.1016/j.mex.2023.102406

**Published:** 2023-10-04

**Authors:** Lorenzo Tavazzani, Jörn-Frederik Wotzlaw, Rita Economos, Dawid Szymanowski, Oscar Laurent, Olivier Bachmann, Cyril Chelle-Michou

**Affiliations:** a Institute of Geochemistry and Petrology, ETH Zürich, Zürich CH-8092, Switzerland; b Department of Earth Sciences, Southern Methodist University, Dallas 75205, USA; c CNRS, Géosciences Environnement Toulouse, Observatoire Midi-Pyrénées, Toulouse F-31400, France

**Keywords:** Zircon, Open-system, Rhyolite-MELTS, Monte Carlo, Granites, Rhyolites, Caldera, Eruption, AgeSpectraAnalyst

## Abstract

In the last decade, improvements in the analytical precision achievable by zircon U-Pb geochronological techniques have allowed to resolve complexities of zircon crystallization histories in magmatic rocks to an unprecedented level. A number of studies have strived to link resolvable dispersion in zircon age spectra of samples from fossil magmatic systems to the physical parameters of their parent magma bodies. However, the methodologies developed have so far been limited to reproduce the effect of simple thermal histories on the final distribution of zircon ages. In this work we take a more nuanced approach, fine-tuning a thermodynamics-based zircon saturation model to predict the relative distribution of zircon ages in samples from silicic magma reservoirs experiencing open-system processes (e.g. heat/mass addition, mechanical mixing). Employing the MATLAB package (AgeSpectraAnalyst) presented in this contribution:

• Users can forward model the effect that diverse thermal histories and mechanical mixing processes characteristic of silicic magma bodies have on zircon age distributions as measured by high-precision, chemical abrasion thermal ionization mass spectrometry (CA-ID-TIMS) U-Pb geochronology.
• Zircon CA-ID-TIMS datasets from silicic magmatic systems can be easily compared with model output to gain semi-quantitative information on thermo-mechanical history of the system of interest.
• We demonstrated (Tavazzani et al., in press) that distribution of high-precision zircon ages in crystallized remnants of shallow (∼ 250 MPa), silicic magma reservoirs can discriminate between systems that experienced catastrophic, caldera-forming eruptions and systems that underwent monotonic cooling histories.

## Graphical abstract

**Figure:**
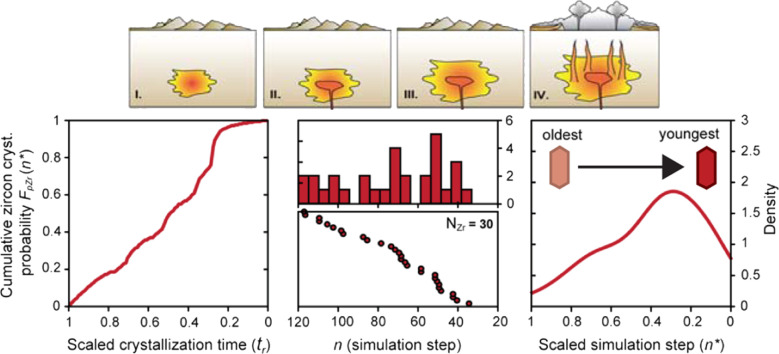

Specifications table

**Table:** 

Subject area:	Earth and Planetary Sciences
More specific subject area:	Igneous petrology and zircon U-Pb geochronology
Name of your method:	AgeSpectraAnalyst
Name and reference of original method:	- [1] Temporal variation in relative zircon abundance throughout Earth history. Geochem. Persp. Let. 179–189. 10.7185/geochemlet.1721 - [2] A stochastic sampling approach to zircon eruption age interpretation. Geochem. Persp. Let. 31–35. 10.7185/geochemlet.1826
Resource availability:	The MATLAB^Ⓡ^ scripts: **StochasticZirconCrystallization.m** **CrystallizationTemperatureEvolution.m** **Resampling_for_mixing.m** are available at https://github.com/TavazzaniL/OpenSystemZrSat along with .csv files containing the vectors $f_{p_{Zr}}\left(n\right)$ obtained from crystallization simulation scenarios listed in Table 2.

## Background

The temporal evolution of magmatic systems can be revealed by analyses of refractory minerals incorporating radiogenic isotopes that decay with time. U-Pb in zircon has long been recognized as the most robust system for this purpose [3] and the improvement of analytical protocols and instrumentation design (e.g. [4], [5], [6], [7]) now allows determination of absolute ages with best-case internal analytical uncertainties better than 0.02 % for bulk-grain analyses of zircon through chemical abrasion-isotope dilution-thermal ionization mass spectrometry (CA-ID-TIMS; [8]). Increase in precision means that for individual samples dispersion of single analyses outside of analytical uncertainty is commonly observed. The analysis of zircon age distributions to reconstruct the magmatic flux in a particular magmatic system is now a well-established branch of zircon petrochronology (e.g. [9], [10], [11], [12]). However, some of the major limitations of this approach are: (1) the modeling of zircon mass distribution at the scale of an entire magmatic system, rather than at the sample scale, inevitably blurring the heterogeneous evolution of incrementally assembled reservoirs; (2) the assumption of a constant zircon crystallization rate during the lifetime of a magmatic system or that (3) this crystallization rate can be parametrized based on theoretical assumptions [13]. In order to circumvent these limitations, some authors have employed thermodynamics-based zircon saturation models, which integrate temperature, crystallinity, major and trace element evolution of a magma batch to assign petrologic meaning to dispersed zircon age populations observed in rock samples (e.g. [1,2,[14], [15], [16]]). So far, these modeling efforts have been successful but have seen their application limited to relatively simple cases of monotonic cooled magma batches. A new generation of models, which include the effect of open-system process on zircon saturation, is needed in order to interpret the relative distribution of zircon mass (i.e. zircon age distributions) in samples collected from the crystalized remnants of long-lived, thermochemically complex magmatic systems.

## Methodology proposed

The zircon saturation modeling presented here is based on the numerical approach developed by Keller et al. [1,2] modified to include non-monotonic cooling histories. This method focuses on calc-alkaline, evolved magma compositions (e.g. granites, rhyolites) as they source most catastrophic, caldera-forming eruptions [17] and their near-ubiquitous zircon saturation [[18], [19], [20]] makes them preferential targets of high-precision zircon geochronology studies (e.g. [[21], [22], [23], [24]]).

### Rhyolite-MELTS zircon crystallization distribution

**Fig. 1:**
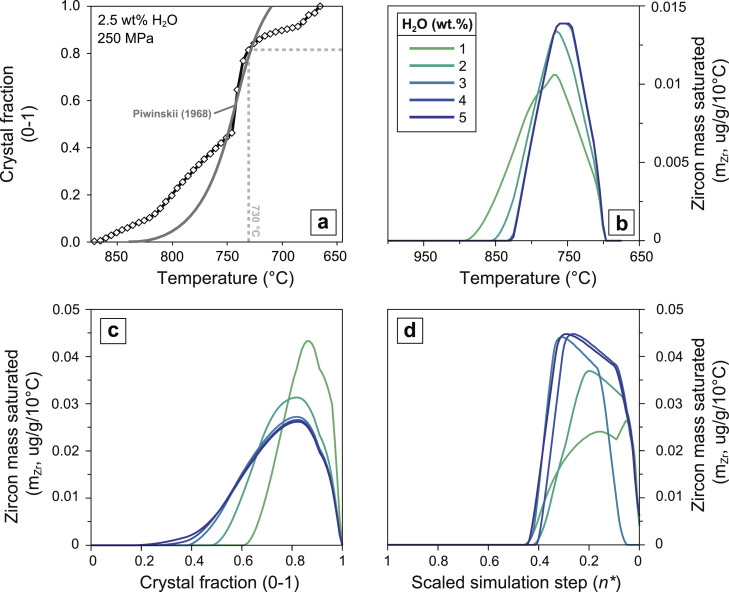
Theoretical and modeling setup for the zircon saturation model. (a) Rhyolite-MELTS derived temperature crystal-fraction curve (i.e. solidus-liquidus relation) for starting granitic composition (Table 1). A best-fit line for crystallization experiments of Piwinskii [33] composition 104 (i.e. biotite granite) at 250 MPa is shown for reference. Normalized mass of saturated zircon (m_Zr_) is plotted vs. (b) temperature, (c) percent of residual melt and (d) normalized number of crystallization simulation steps (n*) for fractional crystallization simulations ran at variable magmatic H_2_O content (1–5 wt%) and starting granitic composition (Table 1).

Model zircon crystallization distributions are calculated from rhyolite-MELTS [25] major-element equilibrium crystallization (EQ), fractional crystallization (FC) and recharge fractional crystallization (RFC) simulations (run on the software Magma Chamber Simulator, https://mcs.geol.ucsb.edu; [26,27]) using the saturation model of Boehnke et al. [28]. A rhyolitic composition (from [29], given in Table 1; Fig. 1) is used as starting melt composition at a pressure of 250 MPa. This pressure corresponds to the optimal depth range for magma chamber nucleation and growth as suggested by physical models [30] and a compilation of natural datasets [31]. Initial magmatic water content is set to 2.5 wt% H_2_O and ƒO_2_ to the QFM (quartz-fayalite-magnetite) buffer. Increase of H_2_O content above 2.5 wt% does not substantially affect the distribution of saturated zircon mass (Fig. 1). The effect on zircon saturation generated by the presence of a mixed volatile magmatic phase (e.g. with additional CO_2_, H_2_S, SO_2_, etc.) is not evaluated in this work. In RFC simulations, rhyolitic and basaltic andesite compositions (also from [29], given in Table 1) are used as liquid compositions of silicic and mafic recharges, respectively. A complete list of crystallization simulations explored in this work is provided in Table 2. Each crystallization simulation is allowed to reach solidus temperature or alternatively is interrupted at 750 °C (*c.* 50 vol.% melt; Fig. 1), to simulate the abrupt termination of crystallization caused by an eruptive event.

**Table 1:** Valle Mosso pluton whole-rock geochemistry used in zircon saturation modeling (for sampling location and petrographic description of PST214 and Campore sample, see respectively [29] and [32]).

Analyte (wt%)	PST214	Campore
SiO2	73.79	49.69
Al2O3	0.20	1.68
FeO (Tot)	14.29	16.22
MnO	1.59	9.74
MgO	0.03	0.16
CaO	0.35	8.69
Na2O	1.12	9.94
K2O	3.27	2.89
TiO2	5.28	0.67
P2O5	0.08	0.32
**Zr (ppm)**	**137**	**136**

**Table 2:** Crystallization simulation scenarios (for compositions refer to Table 1).

Simulation N# - ID	Cryst. scenario	Initial composition	Initial T (°C)	Number recharges	Recharge composition	Recharge T (°C)	Recharge mass (g)
1 - fc	FC	PST214	871	-	-	-	-
2 - 1a2	RFC	PST214	871	1	Campore	1100	10
3 - 1b	RFC	PST214	871	2	Campore	1100	10
4 - 1e	RFC	PST214	871	3	Campore	1100	10
5 - 5a	RFC	PST214	871	4	Campore	1100	10
6 - 5b	RFC	PST214	871	5	Campore	1100	10
7 - 2a	RFC	PST214	871	1	PST214	870	50
8 - 2c	RFC	PST214	871	2	PST214	870	50
9 - 2d	RFC	PST214	871	3	PST214	870	50
10 - 5e	RFC	PST214	871	4	PST214	870	50
11 - 5f	RFC	PST214	871	5	PST214	870	50
12 - 3a	RFC	PST214	871	1	PST214	870	25
13 - 3e	RFC	PST214	871	2	PST214	870	25
14 - 3d	RFC	PST214	871	3	PST214	870	25
15 - 5g	RFC	PST214	871	4	PST214	870	25
16 - 5h	RFC	PST214	871	5	PST214	870	25
17 - 4c	RFC	PST214	871	1	Campore	1100	5
18 - 4d	RFC	PST214	871	2	Campore	1100	5
19 - 4e	RFC	PST214	871	3	Campore	1100	5
20 - 5c	RFC	PST214	871	4	Campore	1100	5
21 - 5d	RFC	PST214	871	5	Campore	1100	5

At each crystallization simulation step the concentration of zirconium in the residual melt fraction (${\left[\text{Zr}\right]}_{\text{calc}}^{\text{melt}}$) is calculated through the Rayleigh equation for fractional crystallization:

(1) $${\left[\text{Zr}\right]}_{\text{calc}}^{\text{melt}}\;=\;F^{\left(D-1\right)}{\left[\text{Zr}\right]}_{\text{initial}}^{\text{melt}}$$

Where F is the melt fraction value output from rhyolite-MELTS, ${\left[Zr\right]}_{initial}^{melt}$ the zirconium concentration in the initial liquid (Table 1), and D the bulk Zr partition coefficient determined using rhyolite-MELTS modal mineral abundances and Zr partition coefficients compiled from the Geochemical Earth Reference Model (see Supplementary Table 1).

The direct relationship between zircon saturation [28], temperature, and melt composition is expressed as:

(2) $${\left[Zr\right]}_{sat}^{melt}\;=\;\frac{496000}{e^{\left[\frac{10108}{T}+1.16\;\left(M-1\right)-1.48\right]}}$$

where the T is temperature (in K, output from rhyolite-MELTS) and M the parameter describing degree of silicate melt polymerization (i.e. the balance between network modifying cations: Na, K, Ca, Mg, Fe, and silicate melt network formers: Si, Al, which affects zirconium solubility in a melt) calculated after Watson and Harrison [18] as:

(3) $$M\;=\;\frac{\left(Na+K+2Ca\right)}{\left(Al\;\times \;Si\right)}$$

where Na, K, Ca, Al, and Si are expressed as molar, normalized element components output from rhyolite-MELTS.

Zircon growth initiates when ${\left[\text{Zr}\right]}_{\text{calc}}^{\text{melt}}\;>\;{\left[\text{Zr}\right]}_{\text{sat}}^{\text{melt}}$ and the mass of zircon crystallized is determined at each simulation interval as:

(4) $$m_{\text{Zr}}=\left\{ \begin{array}{c} m_{\text{Zr}\;}=\;\frac{m_{\text{liq}}}{100\;\left({\left[\text{Zr}\right]}_{\text{calc}}^{\text{melt}}\;-\;{\left[\text{Zr}\right]}_{\text{sat}}^{\text{melt}}\right)}\;\;{\left[\text{Zr}\right]}_{\text{calc}}^{\text{melt}}\;>\;{\left[\text{Zr}\right]}_{\text{sat}}^{\text{melt}}\\ m_{\text{Zr}}\;=\;0\;{\left[\text{Zr}\right]}_{\text{calc}}^{\text{melt}}\;<\;{\left[\text{Zr}\right]}_{\text{sat}}^{\text{melt}} \end{array}\right.$$

where m_liq_ is the mass of liquid phase output from rhyolite-MELTS (in grams). Trivial differences in crystallized zircon mass distributions are observed when tying dependence of zircon saturation on melt temperature and composition (Eq. (2)) with the most recent Crisp and Berry [20] experimental calibration.

**Fig. 2:**
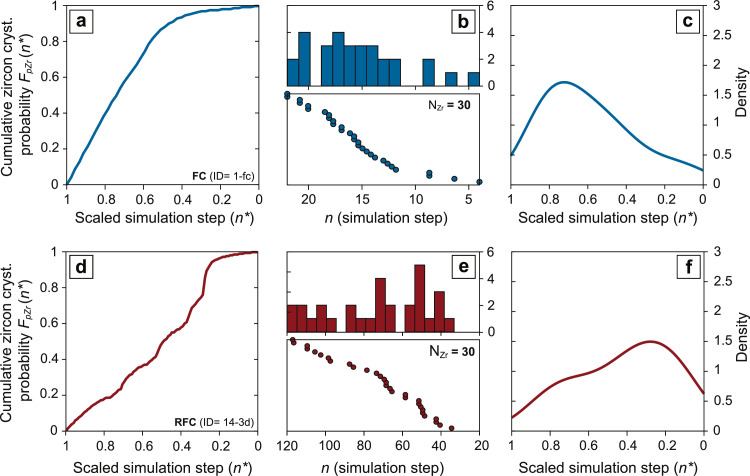
Visualization of the stochastic zircon sampling workflow. Synthetic zircon age and temperature distributions are scaled from initiation (x=1) to termination (x=0) of zircon crystallization. Panels (a) and (d) represent cumulative zircon crystallization probability distribution functions $F_{p_{Zr}}\left(n^{*}\right)$ calculated from crystallization probability density function $f_{p_{Zr}}\left(n\right)$ through Eq. (8). Panels (b) and (e) represent the result of random sampling from $F_{p_{Zr}}\left(n^{*}\right)$, where the 30 randomly selected crystallization steps (n) are shown as frequency histograms (upper boxes) and rank-order plots (lower boxes). In panel (c) and (f), the randomly sampled zircon crystallization distributions are displayed as kernel density estimates of the scaled simulation steps (n*). Plots in (a), (b), (c) correspond to fractional crystallization (simulation ID: 1-fc from Table 2) and (d), (e), (f) recharge fractional crystallization (simulation ID: 14-3d from Table 2) simulations, respectively.

This workflow produces a distribution of zircon growth as a function of temperature and melt fraction in batch crystallization and fractional crystallization simulations (i.e. zircon crystallization spectra, Fig. 1; [1,14]). However, in complex crystallization simulations (RFC), recharge-induced fluctuations of intensive parameters cause a non-linear relationship between mass of zircon crystallized and temperature (or melt fraction), with individual temperature values (or melt fraction values) corresponding to multiple values of crystalized zircon mass. To address this problem, we express the zircon crystallization distribution as a function of the number of steps (n) from crystallization initiation (Fig. 2). In rhyolite-MELTS simulations, if an equal amount of enthalpy (e.g. 1Jg^−1^) is extracted at each step from the magma during solidification [34,35] the number of modelling steps becomes proportional to the time spent by the magma within a given interval of temperature. Under this assumption we thus have a relative crystallization chronology, which provides a time-integrated record of zircon growth/dissolution between each scenario of interest.

### Stochastic sampling of rhyolite-MELTS zircon crystallization distributions

The limited number of analyses per specimen obtained with high-precision geochronological techniques (e.g. CA-ID-TIMS) may skew natural zircon age distributions towards intervals of high zircon crystallization at the expense of relatively low zircon growth periods. To provide a realistic comparison between natural (i.e. CA-ID-TIMS) and synthetic zircon age populations, we developed a workflow that allows the random sampling of an arbitrary number of zircons from a model zircon crystallization probability density function (generated from rhyolite-MELTS simulations following the numerical approach described in the previous section). To this end, the MATLAB^Ⓡ^ script **StochasticZirconCrystallization.m** implements the stochastic sampling of rhyolite-MELTS derived zircon crystallization distribution (listed in Table 2).

The probability of generating a zircon at a particular step (*n*) of a crystallization simulation is defined as:

(5) $$p_{Zr_{n}}=\frac{dm_{Zr_{n}}}{\sum_{i=0}^{N}dm_{Zr_{i}}}$$

where $dm_{Zr_{n}}$ is the relative change in available zircon mass $m_{Zr_{n}}$ (Eq. (4)) between two successive simulation steps:

(6) $$dm_{Zr_{n}}\;=\;\left\{ \begin{array}{c} dm_{Zr_{n}}\;=\;m_{Zr_{n}}\;-\;m_{Zr_{\left(n-1\right)}}\;m_{Zr_{n}\;}-\;m_{Zr_{\left(n-1\right)}}\;>\;0\\ dm_{Zr_{n}}\;=\;0\;\;m_{Zr_{n}}\;-\;m_{Zr_{\left(n-1\right)}}\;\leq \;0 \end{array}\right.$$

which is normalized to the overall variation in crystallized zircon mass ($\sum_{i=0}^{N}dm_{Zr_{i}}$, where N is the total number of steps in a crystallization simulation). As our model does not account for negative zircon growth (i.e. zircon dissolution), any simulation step defined by negative change in zircon mass is set to $dm_{Zr_{n}}=0$.

A zircon crystallization probability density function $f_{p_{Zr}}\left(n\right)$ is thus defined for each simulation, with non-uniform distribution:

(7) $$p_{Zr_{n}}\geq 0\;\text{and}\;\sum_{n=0}^{N}p_{Zr_{n}}=1$$

A number of zircons is randomly sampled from $f_{p_{Zr}}\left(n\right)$ to simulate the stochastic process of zircon selection during geochronological analysis. The cumulative distribution function $F_{p_{Zr}}\left(n\right)$ of the target probability density function $f_{p_{Zr}}\left(n\right)$ is obtained as:

(8) $$F_{p_{Zr}}\left(n\right)=\int_{-\infty }^{n}f_{p_{Zr}}\left(n^{\prime }\right)dn{}^{\prime }$$

and the variable of interest (in this case *n*, a specific crystallization step) is randomly selected from the cumulative distribution function $F_{p_{Zr}}\left(n\right)$ as:

(9) $$n=F_{p_{Zr}}^{-1}\left(X\right)\;\text{where}\;X=F_{p_{Zr}}\left(n\right)$$

**Fig. 3:**
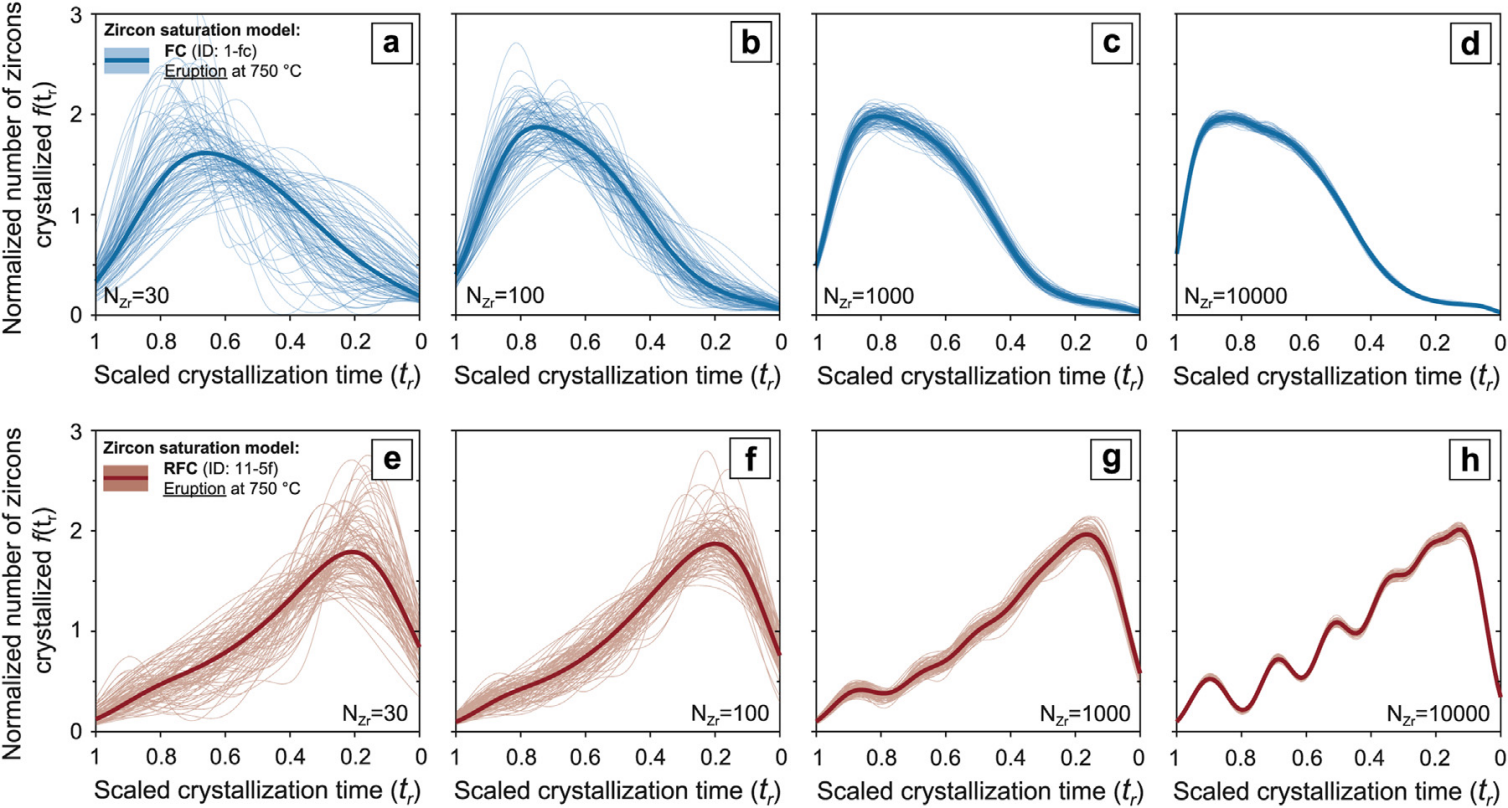
Effect of over- and under-sampling on the shape of synthetic zircon age spectra. Synthetic zircon age and temperature distributions are scaled from initiation (x=1) to termination (x=0) of zircon crystallization. Kernel density estimates (KDEs) of individual and averaged (bold lines) stochastic outputs from (a), (b), (c), (d) fractional crystallization (simulation ID: 1-fc from Table 2) and (e), (f), (g), (h) recharge fractional crystallization (simulation ID: 11-5f from Table 2) simulations. From left to right the number of stochastically sampled zircon crystallization steps (n) per simulation is increased from N_Zr_ = 30 to N_Zr_=10000. The random selection algorithm undergoes 10^3^ iterations for each simulation.

The *n* variables randomly selected from a non-uniform zircon crystallization probability density function $f_{p_{Zr}}\left(n\right)$ correspond to specific simulation steps at which zircons are observed (Fig. 2). This set of equations is implemented using the MATLAB^Ⓡ^ function randsrc().The number of randomly selected *n* variables (N_Zr_) can be defined by the user (Figs. 2, 3). For each simulation, as to ensure robustness of the random selection process, the random selection algorithm undergoes 10^3^ iterations.

### Visualization of model output - zircon crystallization age density functions (natural and synthetic)

Zircon age distributions are expressed as relative zircon crystallization density functions *f_xtal_(t_r_)* (after [2]), where *t_r_* is relative time, scaled from zircon saturation (*t_sat_*) to full plutonic solidification (*t_sol_*) or eruptive truncation (*t_er_*):

(10a) $$t_{r}=\frac{\left(t-t_{sol}\right)}{\Delta t}\;\text{where}\;\Delta t=t_{sat}-t_{sol}$$

(10b) $$t_{r}=\frac{\left(t-t_{er}\right)}{\Delta t}\;\text{where}\;\Delta t=t_{sat}-t_{er}$$

While in natural samples *t_sat_* and *t_sol_* or *t_er_* correspond to absolute radiometric dates, in the case of model zircon distributions *t_sat_* corresponds to the first simulation step in which ${\left[\text{Zr}\right]}_{\text{calc}}^{\text{melt}}\;>\;{\left[\text{Zr}\right]}_{\text{sat}}^{\text{melt}}$ and *t_sol_* or *t_er_* correspond to the simulation step at which the simulation is interrupted.

The form of relative crystallization distributions *f_xtal_(t_r_)* estimated from natural or synthetic data are then visualized through a truncated kernel density estimate of the scaled crystallization times $t_{r_{obs}}$where:

(11) $$t_{r_{obs}}=\frac{t_{obs}-\text{min}\left(t_{obs}\right)}{\text{max}\left(t_{obs}\right)-\text{min}\left(t_{obs}\right)}$$

To produce the kernel density estimate of *f_xtal_(t_r_)* from $t_{r_{obs}}$we use the MATLAB^Ⓡ^ function ksdensity() with a Gaussian kernel and bandwidth determined by Gaussian approximation. The resulting kernel density estimate is truncated at *t_r_* = -0.05 for natural volcanic samples and model *f_xtal_(t_r_)* produced by simulations interrupted at 750°C (i.e. modeling pre-eruptive zircon crystallization). Kernel density estimates are then scaled between x=1, which denotes the initiation of zircon crystallization at *t_sat_* and x=0 the termination of crystallization at *t_sol_* or *t_er_* (Figs. 2, 3). This methodology (first introduced by [2]) allows a reliable estimate of *f_xtal_(t_r_)*, which can reproduce broad fluctuations in the zircon relative crystallization rate, while maintaining the abrupt cutoff in the crystallization distribution that should be caused by an eruption. The kernel density estimates of natural, CA-ID-TIMS datasets represent averaged zircon crystallization distributions of all the samples collected from a single magmatic system. In synthetic datasets, kernel density estimates are obtained for both zircon age distributions from individual stochastic outputs and zircon age distributions averaged over 10^3^ stochastic outputs. This is done to better display the range of possible zircon crystallization distributions and associated kernel density estimates that are obtainable from the same underlying $f_{p_{Zr}}\left(n\right)$ through a stochastic sampling process.

### Zircon crystallization temperature distribution

**Fig. 4:**
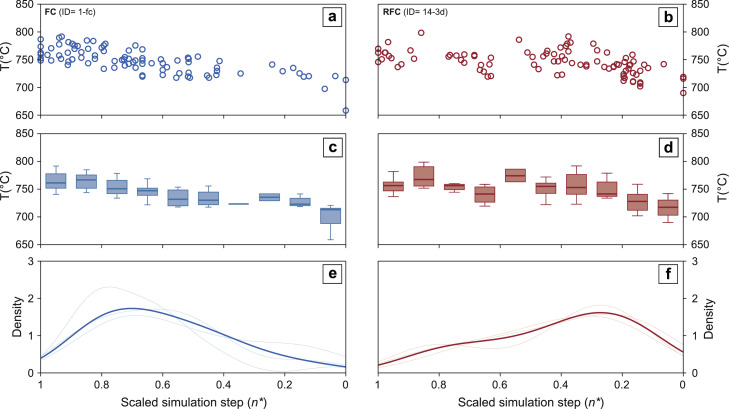
Visualization of zircon crystallization temperature output from stochastic zircon sampling workflow. Individual temperature values that correspond to randomly selected zircon crystallization steps are shown in (a) for a fractional crystallization simulation (ID: 1-fc from Table 2), and for (b) a recharge fractional crystallization simulation (ID: 14-3d from Table 2). (c), (d) Box plots of temperature values from the same simulations. In panel (e) and (f), the randomly sampled zircon crystallization distributions are displayed as kernel density estimates of the scaled simulation steps (n*). Synthetic zircon age and temperature distributions are scaled from initiation (x=1) to termination (x=0) of zircon crystallization. Box plots show median, interquartile ranges (IR) and extreme values for bins corresponding to 1/10 of the total zircon crystallization history.

In the **StochasticZirconCrystallization.m** routine, each simulation step (n) that is randomly selected from a non-uniform zircon crystallization probability density function $f_{p_{Zr}}\left(n\right)$ has a corresponding temperature value. This value represents the temperature of zircon crystallization at a specific simulation step, allowing us to track absolute changes in zircon crystallization temperature in a rhyolitic magma batch, in different crystallization pathways. To take advantage of this perk of the rhyolite-MELTS engine, we implemented an additional routine **[CrystallizationTemperatureEvolution.m]** that returns, in addition to the kernel density estimate of a synthetic zircon population, the distribution of zircon crystallization temperatures for the same simulation (Fig. 4). To account for the analytical uncertainties associated with the application of Ti in zircon thermometry to natural samples (c.f. [36]), the user can add randomized noise (e.g. analytical scatter, uncertainty on titanium activity, on the order of ± 0 to 25 °C; [[11], [37]]) to temperature values obtained for each simulation step.

### Uncertainty parametrization in the generation of CA-ID-TIMS synthetic zircon populations

**Fig. 5:**
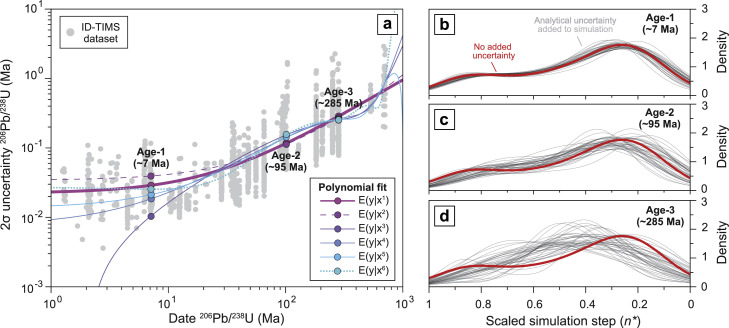
Effect of analytical uncertainty propagation on synthetic zircon age spectra. (a) Least square regressions (LSQs) for a compiled dataset of recently published (2012-2021) CA-ID-TIMS ^206^Pb/^238^U dates and reported 2σ uncertainties. Data are from: [[21], [22], [37], [40], [41], [42], [43], [44], [45], [46], [47], [48], [49], [50], [51], [52], [53], [54], [55], [56], [57], [58], [59], [60], [61], [62], [63], [64], [65]]. Each line represents a different polynomial fit to the dataset, and points on a line corresponding to interpolated 2σ uncertainties for three magmatic systems of increasing crystallization ages (∼ 7 Ma, Capanne pluton, [37]; ∼ 95 Ma, Mt. Stuart pluton, Matzel et al. [66]; ∼285 Ma, Laghi granites, [41]) (b), (c), (d) Visualization of the output of recharge fractional crystallization simulations (simulation ID: 14-3d from Table 2) with added analytical uncertainty corresponding to the three age brackets at 7, 95 and 285 Ma (thin grey lines) compared to an average of 10^2^ iterations for the same crystallization scenario without added uncertainty (bold red line).
(2) Applied a linear regression (least square, LSQ) to the compiled dataset in the interval 1 to 1000 Ma to obtain an empirical relation between ^206^Pb/^238^U age and analytical 2σ uncertainty. As analytical uncertainties in U-Pb dates are symmetric, we only plotted one data point per analysis. Given the spread of the published uncertainties within individual time bins (up to one order of magnitude), the goodness of fit is lacking (r^2^=0.40). Fitting the dataset with higher order polynomial functions does not seem to improve the regression procedure (Fig. 5a). A least square regression of this large dataset provides a first order indication of the evolution of typical analytical uncertainties in CA-ID-TIMS ^206^Pb/^238^U dates with zircon age.
(3) To add the effect of analytical uncertainty corresponding to zircons crystallized at a specific time, the model calculates the median age of the input dataset and uses that value to calculate the characteristic uncertainty from the linear regression (Fig. 5a). A random value picked from the Gaussian uncertainty distribution is then added to each randomly selected zircon date from the cumulative distribution function $F_{p_{Zr}}\left(n\right)$.

To parameterize variation of U-Pb zircon age uncertainties with absolute age of zircon crystallization [38,39] and thus simulating an actual CA-ID-TIMS output, we:

(1) Compiled ∼1700 single-crystal, Th-corrected CA-ID-TIMS zircon ^206^Pb/^238^U dates from 1 to 1000 Ma published between 2011–2021 (Supplementary Table 2; see reference list under Fig. 5) together with their reported analytical uncertainties in Ma (2σ).

The increasing absolute uncertainty with increasing zircon crystallization age (here only ^206^Pb/^238^U ages are discussed) is reflected in the scatter of resampled populations. In simulations of zircon crystallization in younger systems (< 10 Ma) the effect of accounting for analytical uncertainty during probability distribution resampling is nearly insignificant (Fig. 5b). This effect is still very limited in Late Mesozoic magmatic systems (< 100 Ma; Fig. 5c), however it becomes important in simulating zircon age distributions in Early Mesozoic to Paleozoic contexts (Fig. 5d).

## Modeling mechanical mixing of zircon populations

**Fig. 6:**
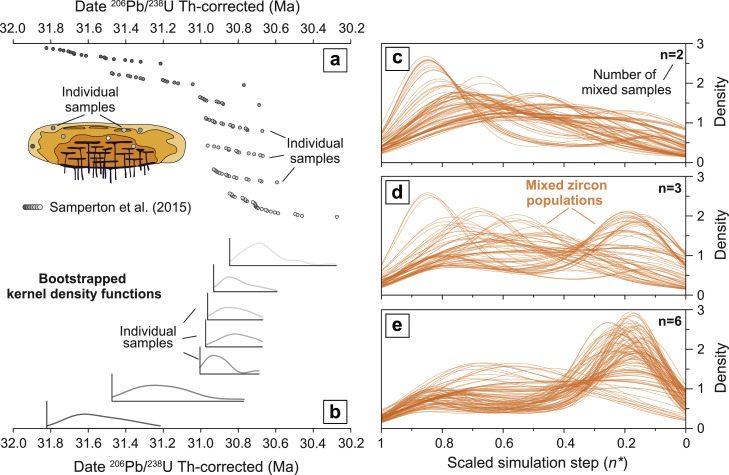
Simulation of zircon age spectra created by mechanical mixing of individual zircon populations. (a) Natural distribution of zircon dates from different samples of the Bergell pluton [68] and (b) bootstrapped zircon age distributions for each one of these samples. The three side panels show the effects of mixing (c) 2, (d) 3 or (e) 6 individual zircon age populations with age distributions identical to that observed in the Bergell samples (thin orange lines, 50 simulations for each panel).

In the run-up to or during a large eruption, mechanical mixing and homogenization of distinct zircon populations (i.e. generated from the cooling of isolated magma batches, or through incorporation of older magmatic products during eruption or transport within a pyroclastic density current; Fig. 6a) can create zircon age populations that do not follow a simple distribution expected from crystallization of a single magma batch. For example, pre-eruptive recharge of an upper crustal magma reservoir may lead to entrainment of various crystallized portions of the system (e.g. [24]). Alternatively, during an eruptive event, the zircons hosted in rocks in proximity of eruptive vents could be taken as crystal-cargo (“chimney sweeping” effect; [67]). The zircon populations that can be created through such mixing will have age distributions that cannot be reproduced with a purely thermodynamic model. Here the stochastic nature of the mechanical mixing process is simulated by juxtaposing and randomly sampling a number of zircon populations that have a parametrized temporal distribution, with an additional MATLAB^Ⓡ^ routine **[Resampling_for_mixing.m]** that allows the simulation of complex mixing of zircon populations:

(1) The model is based on zircon growth curves for a number of temporal intervals (n=7) that correspond to CA-ID-TIMS dated samples of the Bergell pluton (Fig. 6a, b; [68]). The temporal distribution of samples in this intrusion represents a benchmark example of incrementally added magma batches, and nearly every sample shows a zircon age distribution that is expected for monotonic cooling of a magma batch [14].
(2) From a number of zircon populations (user selected) the model creates a cumulative crystallization probability density function $f_{p_{Cum\_Zr}}\left(n\right)$.
(3) The $f_{p_{Cum\_Zr}}\left(n\right)$ is randomly sampled to extract a synthetic zircon population in a similar fashion to that with FC and RFC simulations (previous section), with the user being able to select the number of randomly selected *n* variables (N_Zr_).

The model results show that the number of independent zircon populations involved in the mixing process is key to the final shape of the zircon age distribution. In the case of only two populations sampled in the mechanical mixing process, the output will be very close to the starting (i.e. Bergell samples) zircon distributions (Fig. 6c) with a shape of zircon age spectra similar to that of monotonically cooled magma batches, while a mixing that involves nearly all zircon populations will produce a completely different zircon distribution (Fig. 6e), with an apparent shift in zircon relative abundance.

## Method validation

The chief goal of this method is to gain a better understanding of the genetic processes beyond the diverse zircon age distributions that are observed in granitic and rhyolitic lithologies. To this end, we compare (a) mass spectrometry-derived zircon age distributions from a range of natural samples and (b) stochastically derived zircon age distributions, sampled from synthetic zircon crystallization distributions. As a proof of concept, we selected three long-lived (>1 Myr) silicic magmatic systems (Bergell, [68]; Bingham Canyon, [40]; Sesia, [41,69]) that have been recently characterized both by CA-ID-TIMS geochronology and Ti-in-zircon thermometry.

**Fig. 7:**
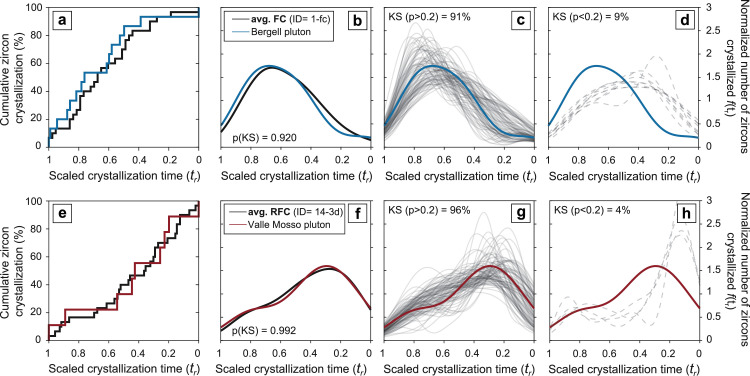
Statistically evaluated performances of two zircon crystallization simulations compared to empirical zircon age distributions from two plutonic systems. Panel (a), (e) display visual comparisons and (b), (f) results of Kolmogorov-Smirnov (KS) test application to cumulative zircon crystallization distributions of averaged stochastic outputs and plutons age spectra. The degree of similarity between model and empirical age spectra is quantified in terms of percent of stochastic outputs that pass (c), (g) or fail (d), (h) a KS test with significance level at 80 % (i.e. p < 0.2). Natural zircon age distributions are from (a), (b), (c), (d) the Bergell pluton (data from [68]) and (e), (f), (g), (h) the Valle Mosso pluton [69].

As a first example, we ran a Kolmogorov-Smirnov (KS; [70]) statistical test to quantify the match between synthetic zircon crystallization density functions (*f_xtal_(t_r_)*) obtained from model zircon crystallization distributions and natural *f_xtal_(t_r_)* from the Bergell [68] and Valle Mosso (Tavazzani et al., in press [69]) intrusive complexes. The KS test determines the probability that two data sets, in this case a synthetic and a natural zircon age distribution, are generated from the same data distribution by comparing their density functions. This test yields an acceptably high KS probability (usually p > 0.05) for both data sets being drawn from the same underlying distribution, whereby the null hypothesis that they are drawn from an identical density function is rejected when p < 0.05 [70,71]. The performance of a modeled crystallization scenario is quantified in terms of percent of stochastic outputs that pass a KS test when compared to empirical zircon age distributions (Fig. 7). In this example, the averaged zircon age distribution of Bergell pluton samples [68] is compared against outputs of a fractional crystallization simulation (Fig. 7a–d) and the average zircon age distribution of the Valle Mosso pluton [69] is evaluated against the synthetic zircon age distributions produced by a recharge fractional crystallization model (Fig. 7e–h). In the case of the Bergell pluton, the good match between simulated fractional crystallization zircon age distributions and measured zircon age distribution (Fig. 7c, d) agrees with overwhelming evidence for a simple, monotonic cooling of the different units of the intrusion [14]. Conversely, the good match between outputs of open-system (RFC) zircon crystallization simulations and the averaged zircon age spectra from the Valle Mosso pluton (Fig. 7g, h) points to a more complex thermal history for this upper crustal intrusion, which likely experienced fluctuations in mass of zircon available for precipitation during its lifetime.

**Fig. 8:**
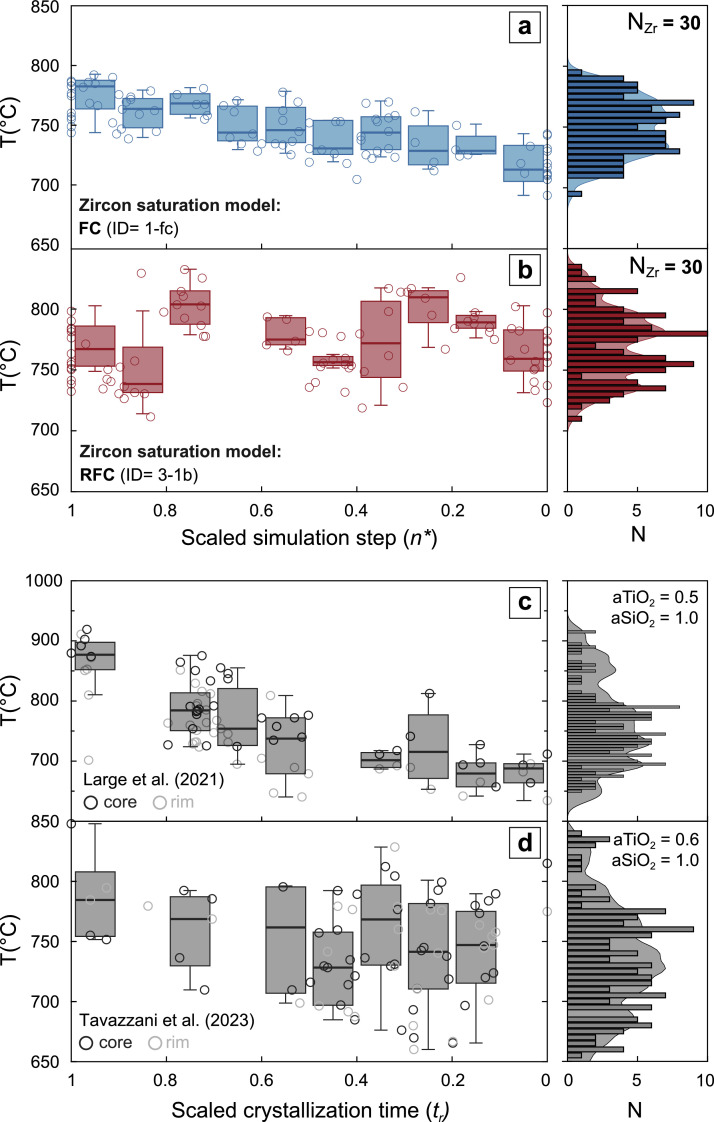
Comparison of synthetic and natural zircon crystallization temperature distributions. Synthetic zircon crystallization temperature distribution patterns obtained from (a) fractional crystallization (simulation ID: 1-fc from Table 2) and (b) recharge fraction crystallization (simulation ID: 3-1b from Table 2) scenarios. Two contrasting natural zircon crystallization temperature distributions are also shown: (c) Bingham Canyon intrusion [40] and (d) Sesia Magmatic System [69]. To allow direct comparison, natural and synthetic data are scaled from initiation (x=1) to termination (x=0) of zircon crystallization. Box plots show median, interquartile ranges (IR) and extreme values for bins corresponding to 1/10 of the total zircon crystallization history.

Secondly, we compared synthetic zircon crystallization temperature evolution obtained for open and closed system crystallization scenarios to the time-resolved record of temperature-modulated trace element concentration (Ti-in-zircon; [72]; Fig. 8) measured in natural zircon populations (e.g. [24,40]). Monotonically decreasing zircon crystallization temperatures characteristic of FC simulations (Fig. 8a) agree with observed zircon temperature distributions in natural systems characterized by emplacement and monotonic cooling of an evolved magma body at upper crustal conditions (e.g. Bingham Canyon intrusion, [40]; Fig. 8c). Conversely, the convergence between systematic fluctuations in crystallization temperatures obtained from RFC simulations (Fig. 8b) and Ti-in-zircon temperature evolution observed across the Valle Mosso pluton (upon application of appropriate activity parameters: a_TiO2_=0.6, a_SiO2_=1.0; from [29]; Fig. 8d), suggest a complex history with open system processes (i.e. recharge) dominant during the lifetime of this reservoir.

## Method strengths and limitations

In contrast to other studies (e.g. [10,12]), the model presented here is effectively adimensional (i.e. 0D). It does not provide absolute temporal constraints on the crystallization process and does not contribute information about the geometry of the system or its spatial heterogeneities. The adimensional aspect of our model with respect to absolute crystallization time is exploited to compare saturation modeling results to zircon age spectra from systems of various sizes and operating over different timescales. In MELTS-based simulations, if an equal amount of enthalpy (e.g., 1 Jg^−1^) is extracted at each step from magma during crystallization, then the number of modeling steps becomes proportional to the time spent by the magma within a given temperature interval [34,35]. Under this assumption, a relative crystallization chronology of zircon mass distributions is obtained in our saturation simulations.

In the saturation model, crystallizing zircons are assumed to be in equilibrium with the surrounding melt at every step of the simulation. We have not included a quantification of the effect that kinetic, disequilibrium processes (i.e. diffusion) have on zircon growth and stability [73]. Although the role of dissolution [74] is neglected, we can qualitatively discuss the effect that transient zircon undersaturation has on the temporal distribution of zircon mass in a periodically recharged magmatic system. Overall, the net effect of periodic zircon undersaturation in RFC scenarios is to bolster the relative zircon crystallization peak close to the end of the magmatic life cycle, through partial loss of early crystallized zircon mass. An exception presents itself when undersaturation can effectively dissolve all early crystallized zircons. In this case, the shape of zircon age spectra will reflect the dominant crystallization process operating in the system after the last magmatic recharge, thus corresponding to an incomplete record of zircon crystallization. However, this occurrence should be rarely observed in evolved subalkaline melts (*sensu* [75]). In melts of this chemical affinity, zircon saturation happens at relatively low Zr concentrations and remelting of zircon-bearing, early cumulate phases during recharge events tends to perpetuate the saturation behavior [76]. Only substantial additions of mafic to intermediate melts, which are rare or subtle in erupted and intrusive products of large silicic systems (e.g. [77]), can produce significant undersaturation. On account of this, results of the saturation model presented in this work should only be applied to evolved, subalkaline systems (e.g. metaluminous and peraluminous rhyolites) that ensure long-term preservation of zircon crystals throughout the recharge and remelting cycles experienced by an upper-crustal magma reservoir [76].

Finally, the effect of competition between growth of existing crystals and nucleation during intervals of zirconium oversaturation [74] is not included in our model. All the zircon mass available for precipitation contributes to crystallization of new zircon grains (i.e. varying nucleation rates but instantaneous growth; [78]). Moreover, in natural datasets, the inherent mixing of age domains within whole grains produced by CA-ID-TIMS analyses may result in slightly shorter crystallization ranges than expected (i.e. averaging effect; [22,68,78]), adding a degree of uncertainty in comparisons between time-resolved natural and synthetic zircon crystallization temperature histories (Fig. 8).

## Concluding remark

The method developed in this study allows users to simulate zircon crystallization trajectories in upper crustal magma reservoirs for a variety of thermal histories. The outputs of this model are tailored to allow a direct comparison with high-precision U-Pb zircon geochronology (CA-ID-TIMS) datasets. Specifically, the stochastic sampling of synthetic zircon age populations mimics the random process of selection of a limited number of zircons from a rock sample, while the uncertainty on the model output is parametrized based on a comprehensive dataset of high-precision zircon ages. The model is inclusive of statistical tools that allow the user to quantitatively compare simulation results and data from natural magmatic systems, thus informing on the dominant thermomechanical processes taking place in a crystallizing magma batch. The provided set of MATLAB^Ⓡ^ codes contains the set of instructions necessary to run any set of simulations, as well as inputting new high-precision zircon datasets to be compared with the various model outputs.

## Ethics statements

This study does not involve any human subjects or animal experiments.

## CRediT authorship contribution statement

**Lorenzo Tavazzani:** Conceptualization, Investigation, Methodology, Software, Visualization, Writing – original draft. **Jörn-Frederik Wotzlaw:** Investigation, Validation, Resources, Writing – review & editing. **Rita Economos:** Supervision, Resources, Writing – review & editing. **Dawid Szymanowski:** Formal analysis, Writing – review & editing. **Oscar Laurent:** Formal analysis, Writing – review & editing. **Olivier Bachmann:** Supervision, Writing – review & editing. **Cyril Chelle-Michou:** Resources, Data curation, Supervision, Writing – review & editing.

## Declaration of Competing Interest

The authors declare that they have no known competing financial interests or personal relationships that could have appeared to influence the work reported in this paper.

## Data Availability

Data and code presented in the study methods will be freely available to any interested third party.

## Appendix Supplementary materials

Supplementary Table 1 - Table including zirconium partition coefficients used to calculate zirconium content in the residual melt fraction. Image, application 1

Supplementary Table 2 – Table including a compilation of ∼1700 single-crystal, Th-corrected CA-ID-TIMS zircon ^206^Pb/^238^U dates from 1 to 1000 Ma with associated analytical uncertainties (2σ), published between 2011-2021. Image, application 2

## References

[1] Keller C.B., Boehnke P., Schoene B. (2017). Temporal variation in relative zircon abundance throughout earth history. Geochem. Perspect. Lett..

[2] Keller C.B., Schoene B., Samperton K.M. (2018). A stochastic sampling approach to zircon eruption age interpretation. Geochem. Perspect. Lett..

[3] Schoene B., Baxter E.F. (2017). Petrochronology and TIMS. Rev. Mineral. Geochem..

[4] Schoene B., Latkoczy C., Schaltegger U., Günther D. (2010). A new method integrating high-precision U–Pb geochronology with zircon trace element analysis (U–Pb TIMS-TEA). Geochim. Cosmochim. Acta.

[5] von Quadt A., Wotzlaw J.F., Buret Y., Large S.J.E., Peytcheva I., Trinquier A. (2016). High-precision zircon U/Pb geochronology by ID-TIMS using new 10 ^13^ ohm resistors. J. Anal. At. Spectrom..

[6] Wotzlaw J.F., Buret Y., Large S.J.E., Szymanowski D., von Quadt A. (2017). ID-TIMS U–Pb geochronology at the 0.1‰ level using 10 13 Ω resistors and simultaneous U and 18 O/16 O isotope ratio determination for accurate UO 2 interference correction. J. Anal. At. Spectrom..

[7] Szymanowski D., Schoene B. (2020). U–Pb ID-TIMS geochronology using ATONA amplifiers. J. Anal. At. Spectrom..

[8] Schaltegger U., Ovtcharova M., Gaynor S.P., Schoene B., Wotzlaw J.F., Davies J.F.H.L., Farina F., Greber N.D., Szymanowski D., Chelle-Michou C. (2021). Long-term repeatability and interlaboratory reproducibility of high-precision ID-TIMS U–Pb geochronology. J. Anal. At. Spectrom..

[9] Caricchi L., Simpson G., Schaltegger U. (2014). Zircons reveal magma fluxes in the Earth's crust. Nature.

[10] Tierney C.R., Schmitt A.K., Lovera O.M., de Silva S.L. (2016). Voluminous plutonism during volcanic quiescence revealed by thermochemical modeling of zircon. Geology.

[11] Kent A.J.R., Cooper K.M. (2018). How well do zircons record the thermal evolution of magmatic systems?. Geology.

[12] Weber G., Caricchi L., Arce J.L., Schmitt A.K. (2020). Determining the current size and state of subvolcanic magma reservoirs. Nat. Commun..

[13] Harrison T.M., Watson E.B., Aikman A.B. (2007). Temperature spectra of zircon crystallization in plutonic rocks. Geol.

[14] Samperton K.M., Bell E.A., Barboni M., Keller C.B., Schoene B. (2017). Zircon age-temperature-compositional spectra in plutonic rocks. Geology.

[15] Ratschbacher B.C., Keller C.B., Schoene B., Paterson S.R., Anderson J.L., Okaya D., Putirka K., Lippoldt R. (2018). A new workflow to assess emplacement duration and melt residence time of compositionally diverse magmas emplaced in a sub-volcanic reservoir. J. Petrol..

[16] Kirkland C.L., Yakymchuk C., Olierook H.K.H., Hartnady M.I.H., Gardiner N.J., Moyen J.F., Hugh Smithies R., Szilas K., Johnson T.E. (2021). Theoretical versus empirical secular change in zircon composition. Earth Planet. Sci. Lett..

[17] Sparks R.S.J., Annen C., Blundy J.D., Cashman K.V., Rust A.C., Jackson M.D. (2019). Formation and dynamics of magma reservoirs. Philos. Trans. R. Soc. Math. Phys. Eng. Sci..

[18] Watson E.B., Harrison T.M. (1983). Zircon saturation revisited: temperature and composition effects in a variety of crustal magma types. Earth Planet. Sci. Lett..

[19] Marxer F., Ulmer P. (2019). Crystallisation and zircon saturation of calc-alkaline tonalite from the Adamello Batholith at upper crustal conditions: an experimental study. Contrib. Mineral. Petrol..

[20] Crisp L.J., Berry A.J. (2022). A new model for zircon saturation in silicate melts. Contrib. Mineral. Petrol..

[21] Wotzlaw J.F., Schaltegger U., Frick D.A., Dungan M.A., Gerdes A., Günther D. (2013). Tracking the evolution of large-volume silicic magma reservoirs from assembly to supereruption. Geology.

[22] Rioux M., Farmer G.L., Bowring S.A., Wooton K.M., Amato J.M., Coleman D.S., Verplanck P.L. (2016). The link between volcanism and plutonism in epizonal magma systems; high-precision U–Pb zircon geochronology from the Organ Mountains caldera and batholith, New Mexico. Contrib. Mineral. Petrol..

[23] Szymanowski D., Wotzlaw J.F., Ellis B.S., Bachmann O., Guillong M., von Quadt A. (2017). Protracted near-solidus storage and pre-eruptive rejuvenation of large magma reservoirs. Nat. Geosci..

[24] Szymanowski D., Ellis B.S., Wotzlaw J.F., Bachmann O. (2019). Maturation and rejuvenation of a silicic magma reservoir: high-resolution chronology of the Kneeling Nun Tuff. Earth Planet. Sci. Lett..

[25] Gualda G.A.R., Ghiorso M.S., Lemons R.V., Carley T.L. (2012). Rhyolite-MELTS: a modified calibration of MELTS optimized for silica-rich, fluid-bearing magmatic systems. J. Petrol..

[26] Bohrson W.A., Spera F.J., Ghiorso M.S., Brown G.A., Creamer J.B., Mayfield A. (2014). Thermodynamic model for energy-constrained open-system evolution of crustal magma bodies undergoing simultaneous recharge, assimilation and crystallization: the magma chamber simulator. J. Petrol..

[27] Bohrson W.A., Spera F.J., Heinonen J.S., Brown G.A., Scruggs M.A., Adams J.V., Takach M.K., Zeff G., Suikkanen E. (2020). Diagnosing open-system magmatic processes using the magma chamber simulator (MCS): part I—major elements and phase equilibria. Contrib. Mineral. Petrol..

[28] Boehnke P., Watson E.B., Trail D., Harrison T.M., Schmitt A.K. (2013). Zircon saturation re-revisited. Chem. Geol..

[29] Tavazzani L., Peres S., Sinigoi S., Demarchi G., Economos R.C., Quick J.E. (2020). Timescales and mechanisms of crystal-mush rejuvenation and melt extraction recorded in permian plutonic and volcanic rocks of the sesia magmatic system (Southern Alps, Italy). J. Petrol..

[30] Huber C., Townsend M., Degruyter W., Bachmann O. (2019). Optimal depth of subvolcanic magma chamber growth controlled by volatiles and crust rheology. Nat. Geosci..

[31] Gualda G.A.R., Ghiorso M.S. (2013). Low-pressure origin of high-silica rhyolites and granites. J. Geol..

[32] Sinigoi S., Quick J.E., Demarchi G., Klötzli U.S. (2016). Production of hybrid granitic magma at the advancing front of basaltic underplating: Inferences from the Sesia Magmatic System (south-western Alps, Italy). Lithos.

[33] Piwinskii A.J. (1968). Experimental studies of igneous rock series central sierra nevada Batholith, California. J. Geol..

[34] Gualda G.A.R., Gravley D.M., Connor M., Hollmann B., Pamukcu A.S., Bégué F., Ghiorso M.S., Deering C.D. (2018). Climbing the crustal ladder: Magma storage-depth evolution during a volcanic flare-up. Sci. Adv..

[35] Hartung E., Weber G., Caricchi L. (2019). The role of H2O on the extraction of melt from crystallising magmas. Earth Planet. Sci. Lett..

[36] Siégel C., Bryan S.E., Allen C.M., Gust D.A. (2018). Use and abuse of zircon-based thermometers: A critical review and a recommended approach to identify antecrystic zircons. Earth Sci. Rev..

[37] Barboni M., Annen C., Schoene B. (2015). Evaluating the construction and evolution of upper crustal magma reservoirs with coupled U/Pb zircon geochronology and thermal modeling: a case study from the Mt. Capanne pluton (Elba, Italy). Earth Planet. Sci. Lett..

[38] Chiaradia M., Schaltegger U., Spikings R., Wotzlaw J.F., Ovtcharova M. (2013). How accurately can we date the duration of magmatic-hydrothermal events in porphyry systems?–an invited paper. Econ. Geol..

[39] Chelle-Michou C., Schaltegger U., Huston D., Gutzmer J. (2023). Isotopes in Economic Geology, Metallogenesis and Exploration.

[40] Large S.J.E., Buret Y., Wotzlaw J.F., Karakas O., Guillong M., von Quadt A., Heinrich C.A. (2021). Copper-mineralised porphyries sample the evolution of a large-volume silicic magma reservoir from rapid assembly to solidification. Earth Planet. Sci. Lett..

[41] Karakas O., Wotzlaw J.F., Guillong M., Ulmer P., Brack P., Economos R., Bergantz G.W., Sinigoi S., Bachmann O. (2019). The pace of crustal-scale magma accretion and differentiation beneath silicic caldera volcanoes. Geology.

[42] Aguirre-Urreta B., Lescano M., Schmitz M.D., Tunik M., Concheyro A., Rawson P.F., Ramos V.A. (2015). Filling the gap: new precise Early Cretaceous radioisotopic ages from the Andes. Geol. Mag..

[43] Keeley J.A., Link P.K., Fanning C.M., Schmitz M.D. (2013). Pre- to synglacial rift-related volcanism in the Neoproterozoic (Cryogenian) Pocatello Formation, SE Idaho: New SHRIMP and CA-ID-TIMS constraints. Lithosphere.

[44] Klein B.Z., Jagoutz O., Ramezani J. (2021). High-precision geochronology requires that ultrafast mantle-derived magmatic fluxes built the transcrustal Bear Valley Intrusive Suite, Sierra Nevada, California, USA. Geology.

[45] Laurent O., Björnsen J., Wotzlaw J.F., Bretscher S., Pimenta Silva M., Moyen J.F., Ulmer P., Bachmann O. (2020). Earth's earliest granitoids are crystal-rich magma reservoirs tapped by silicic eruptions. Nat. Geosci..

[46] Rivera T.A., Schmitz M.D., Jicha B.R., Crowley J.L. (2016). Zircon petrochronology and 40 Ar/39 Ar sanidine dates for the mesa falls tuff: crystal-scale records of magmatic evolution and the short lifespan of a large yellowstone magma chamber. J. Petrol..

[47] Singer B.S., Jicha B.R., Condon D.J., Macho A.S., Hoffman K.A., Dierkhising J., Brown M.C., Feinberg J.M., Kidane T. (2014). Precise ages of the réunion event and huckleberry ridge excursion: episodic clustering of geomagnetic instabilities and the dynamics of flow within the outer core. Earth Planet. Sci. Lett..

[48] Wall C.J., Hanson R.E., Schmitz M., Price J.D., Donovan R.N., Boro J.R., Eschberger A.M., Toews C.E. (2021). Integrating zircon trace-element geochemistry and high-precision U-Pb zircon geochronology to resolve the timing and petrogenesis of the late Ediacaran–Cambrian Wichita igneous province, Southern Oklahoma Aulacogen, USA. Geology.

[49] Burgess S.D., Bowring S.A. (2015). High-precision geochronology confirms voluminous magmatism before, during, and after Earth's most severe extinction. Sci. Adv..

[50] Smyth H.R., Crowley Q.G., Hall R., Kinny P.D., Hamilton P.J., Schmidt D.N. (2011). A toba-scale eruption in the early miocene: the semilir eruption, East Java, Indonesia. Lithos.

[51] Leuthold J., Müntener O., Baumgartner L.P., Putlitz B., Ovtcharova M., Schaltegger U. (2012). Time resolved construction of a bimodal laccolith (Torres del Paine, Patagonia). Earth Planet. Sci. Lett..

[52] Wotzlaw J.F., Bindeman I.N., Schaltegger U., Brooks C.K., Naslund H.R. (2012). High-resolution insights into episodes of crystallization, hydrothermal alteration and remelting in the Skaergaard intrusive complex. Earth Planet. Sci. Lett..

[53] Wotzlaw J.F., Bindeman I.N., Watts K.E., Schmitt A.K., Caricchi L., Schaltegger U. (2014). Linking rapid magma reservoir assembly and eruption trigger mechanisms at evolved Yellowstone-type supervolcanoes. Geology.

[54] Wotzlaw J.F., Bindeman I.N., Stern R.A., D'Abzac F.X., Schaltegger U. (2015). Rapid heterogeneous assembly of multiple magma reservoirs prior to Yellowstone supereruptions. Sci. Rep..

[55] Chelle-Michou C., Chiaradia M., Ovtcharova M., Ulianov A., Wotzlaw J.F. (2014). Zircon petrochronology reveals the temporal link between porphyry systems and the magmatic evolution of their hidden plutonic roots (the Eocene Coroccohuayco deposit, Peru). Lithos.

[56] Schoene B., Samperton K.M., Eddy M.P., Keller G., Adatte T., Bowring S.A., Khadri S.F.R., Gertsch B. (2015). U-Pb geochronology of the Deccan Traps and relation to the end-cretaceous mass extinction. Science.

[57] Zeh A., Ovtcharova M., Wilson A.H., Schaltegger U. (2015). The Bushveld Complex was emplaced and cooled in less than one million years – results of zirconology, and geotectonic implications. Earth Planet. Sci. Lett..

[58] Buret Y., von Quadt A., Heinrich C., Selby D., Wälle M., Peytcheva I. (2016). From a long-lived upper-crustal magma chamber to rapid porphyry copper emplacement: reading the geochemistry of zircon crystals at Bajo de la Alumbrera (NW Argentina). Earth Planet. Sci. Lett..

[59] Burgess S.D., Bowring S.A., Fleming T.H., Elliot D.H. (2015). High-precision geochronology links the ferrar large igneous province with early-Jurassic ocean anoxia and biotic crisis. Earth Planet. Sci. Lett..

[60] Eddy M.P., Bowring S.A., Miller R.B., Tepper J.H. (2016). Rapid assembly and crystallization of a fossil large-volume silicic magma chamber. Geology.

[61] Reimink J.R., Davies J.H.F.L., Chacko T., Stern R.A., Heaman L.M., Sarkar C., Schaltegger U., Creaser R.A., Pearson D.G. (2016). No evidence for Hadean continental crust within Earth's oldest evolved rock unit. Nat. Geosci..

[62] Manor M.J., Scoates J.S., Wall C.J., Nixon G.T., Friedman R.M., Amini M., Ames D.E. (2017). Age of the late cretaceous ultramafic-hosted giant mascot Ni-Cu-PGE deposit, Southern Canadian Cordillera: integrating CA-ID-TIMS and LA-ICP-MS U-Pb geochronology and trace element geochemistry of zircon*. Econ. Geol..

[63] Farina F., Dini A., Davies J.H.F.L., Ovtcharova M., Greber N.D., Bouvier A.S., Baumgartner L., Ulianov A., Schaltegger U. (2018). Zircon petrochronology reveals the timescale and mechanism of anatectic magma formation. Earth Planet. Sci. Lett..

[64] Lukács R., Harangi S., Guillong M., Bachmann O., Fodor L., Buret Y., Dunkl I., Sliwinski J., von Quadt A., Peytcheva I., Zimmerer M. (2018). Early to mid-miocene syn-extensional massive silicic volcanism in the pannonian basin (East-Central Europe): eruption chronology, correlation potential and geodynamic implications. Earth Sci. Rev..

[65] Schaen A.J., Schoene B., Dufek J., Singer B.S., Eddy M.P., Jicha B.R., Cottle J.M. (2021). Transient rhyolite melt extraction to produce a shallow granitic pluton. Sci. Adv..

[66] Matzel J.E.P., Bowring S.A., Miller R.B. (2006). Time scales of pluton construction at differing crustal levels: examples from the Mount Stuart and Tenpeak intrusions, North Cascades, Washington. Geol. Soc. Am. Bull..

[67] Curry A., Gaynor S.P., Davies J.H.F.L., Ovtcharova M., Simpson G., Caricchi L. (2021). Timescales and thermal evolution of large silicic magma reservoirs during an ignimbrite flare-up: perspectives from zircon. Contrib. Mineral. Petrol..

[68] Samperton K.M., Schoene B., Cottle J.M., Brenhin Keller C., Crowley J.L., Schmitz M.D. (2015). Magma emplacement, differentiation and cooling in the middle crust: integrated zircon geochronological–geochemical constraints from the bergell intrusion, central Alps. Chem. Geol..

[69] Tavazzani L., Wotzlaw J.F., Economos R.C., Sinigoi S., Demarchi G., Szymanowski D., Laurent O., Bachmann O., Chelle-Michou C. (2023). High-precision zircon age spectra record the dynamics and evolution of open-system, large silicic magma reservoirs. Earth Planet. Sci. Lett..

[70] Press W.H., Flannery B.P., Teukolsky S., Vetterling W.T. (1988).

[71] Fletcher J.M., Grove M., Kimbrough D., Lovera O., Gehrels G.E. (2007). Ridge-trench interactions and the neogene tectonic evolution of the Magdalena shelf and southern Gulf of California: insights from detrital zircon U-Pb ages from the Magdalena fan and adjacent areas. Geol. Soc. Am. Bull..

[72] Ferry J.M., Watson E.B. (2007). New thermodynamic models and revised calibrations for the Ti-in-zircon and Zr-in-rutile thermometers. Contrib. Mineral. Petrol..

[73] Watson E.B. (1996). Dissolution, growth and survival of zircons during crustal fusion: kinetic principals, geological models and implications for isotopic inheritance. Earth Environ. Sci. Trans. R. Soc. Edinb..

[74] Bindeman I.N., Melnik O.E. (2016). Zircon survival, rebirth and recycling during crustal melting, magma crystallization, and mixing based on numerical modelling. J. Petrol..

[75] Irvine T.N., Baragar W.R.A. (1971). A guide to the chemical classification of the common volcanic rocks. Can. J. Earth Sci..

[76] Szymanowski D., Forni F., Wolff J.A., Ellis B.S. (2020). Modulation of zircon solubility by crystal–melt dynamics. Geology.

[77] Bachmann O., Dungan M.A., Lipman P.W. (2002). The fish canyon magma body, San Juan Volcanic Field, Colorado: rejuvenation and eruption of an upper-crustal batholith. J. Petrol..

[78] Klein B.Z., Eddy M.P. (2023). What’s in an age? Calculation and interpretation of ages and durations from U-Pb zircon geochronology of igneous rocks. GSA Bullet..

